# Circular RNA SNX27 Facilitates Gastric Cancer Progression By Sponging miR-638

**DOI:** 10.5152/tjg.2024.23178

**Published:** 2024-04-01

**Authors:** Yanfeng Xi, Yu Gai, Weixing Zhang, Meihua Wang, Qian Liu, Yanzhi Bi

**Affiliations:** 1 Department of Gastrointestinal Surgery, Changzhou Cancer Hospital, Changzhou, Jiangsu, China; 2 Department of Pathology, Changzhou Cancer Hospital, Changzhou, Jiangsu, China; 3 Department of Clinical Laboratory, Changzhou Cancer Hospital, Changzhou, Jiangsu, China; 4 Department of Oncology, Changzhou Cancer Hospital, Changzhou, Jiangsu, China

**Keywords:** circSNX27, gastric cancer, miR-638

## Abstract

**Background/Aims:**

Accumulating evidences have shown an important role of circular RNAs (circRNAs) in the tumorigenesis of gastric cancer (GC). Nevertheless, whether circSNX27 plays a role in GC remains undetermined.

**Materials and Methods:**

Relative expression of circRNAs and related microRNAs (miRNAs) in GC tissues and cells were tested by quantitative reverse transcription polymerase chain reaction. Specific short hairpin RNAs were designed to knockdown the expression of circSNX27 in GC cells. CCK-8, colony formation, flow cytometry, wound healing, and transwell assays were used to access the function of circSNX27 silencing on GC cells. The target miRNAs of circSNX27 were predicted by 2 databases, circBank and Circinteractome. Dual-luciferase reporter assay was used to verify the interaction between circSNX27 and miR-638.

**Results:**

circSNX27 was found to be upregulated in GC tissues and cell lines compared with normal controls. Silencing of circSNX27 repressed GC cell viability, proliferation, migration, and invasion. Moreover, circSNX27 silencing could accelerate GC cell apoptosis. Additionally, we found that circSXN27 decreased the expression of miR-638 by directly binding to it in GC cells.

**Conclusion:**

Our results indicated that circSXN27 facilitated GC progression by acting as a sponge of miR-638.

**Main Points**

- circSNX27 is elevated in gastric cancer (GC).
- Knockdown of circSNX27 represses GC progression.
- circSNX27 acts as a sponge of miR-638 in GC.

## Introduction

Gastric cancer (GC) was the fourth most frequent solid cancer and the second leading cause of tumor-related death worldwide.^1,2^ The prevalence and mortality rates of GC are particularly high in East Asian countries, where over 60% of new GC cases have been reported in China, South Korea, and Japan.^3^ In recent decades, the overall GC survival rate has improved worldwide due to improvements in early diagnosis and therapeutic strategies.^4^ Nevertheless, the age-standardized 5-year GC survival rate is still as low as 20%-40% in many countries, according to an investigation of the global surveillance of trends in cancer programs.^5^ Molecular diversity leads to high clinical heterogeneity of GC even with similar clinical and pathological features, which increases the difficulty of GC treatment.^6^ A better understanding of the potential molecular mechanisms of cancer progression is essential to develop personalized treatment.

Circular RNAs (circRNAs) are a unique subgroup of noncoding RNAs that are not normally involved in protein coding.^7^ However, their abundance, conservation in evolution, high stability, and cell-type or tissue-specific expression patterns endow them a variety of critical biological functions.^7^ A large number of clinical trials have shown that circRNAs can be used as promising biomarkers for cancer screening, diagnosis, and prognosis assessment.^8,9^ They act as an important regulator of a variety of human cancers by sponging microRNAs, forming RNA–protein complexes, and modulating gene splicing/transcription.^10^ Based on the annotation of circBase website, a total of 22 human circRNA isoforms were generated from the sorting nexin 27 (SNX27) gene. Among these circRNAs, circSNX27, also named as circ_100338 (circBase No. Has_circ_0000130), was reported to be elevated in the tissues of hepatocellular carcinoma (HCC) and contributes to the development of HCC.^11^ However, whether circSNX27 plays a role in GC remains undetermined.

In this study, we aimed to explore the cell function and the potential mechanism of circSNX27 in GC. We first tested the expression of circSNX27 in GC tissues and found a significant elevation of circSNX27 in GC. Then, we silenced its expression in GC cell line and assessed the knockdown impacts on GC cell viability, proliferation, apoptosis, invasion, and migration. Finally, the potential mechanism of circSNX27 was explored.

## Materials and Methods

### Human Gastric Cancer Tissue Collection

Nine pairs of human gastric adenocarcinoma and corresponding normal tissues were gathered from GC patients who received resection surgery in Cahngzhou Cancer Hospital, Changzhou, Jiangsu, China. Tissues were kept in liquid nitrogen until use. Consents were obtained from all patients prior to the study. The study was approved by the Ethics Committee of Changzhou Cancer Hospital (2022 (SR) No. 024). All the experiments were carried out in accordance with the Code of Ethics of the World Medical Association (Declaration of Helsinki).

### Cell Culture

Human gastric adenocarcinoma cell lines, HGC27, AGS, MKN45, MGC803, and SGC7901, as well as GES-1 (a normal human gastric epithelial cell line), were all provided by the American Type Culture Collection (ATCC, Rockefeller, Maryland, USA). Then, all these cells were cultured in Dulbecco’s Modified Eagle’s Medium (DMEM) (Gibco, Carlsbad, California, USA) containing 10% fetal bovine serum (FBS) and placed under 5% CO_2_ atmosphere.

### Quantitative Real-Time Polymerase Chain Reaction Analysis

Total RNAs were extracted using TRIzol reagent (Invitrogen, Carlsbad, California, USA). 5 µg RNA was subjected for complementary DNA synthesis with the help of a Prime-Script reverse-transcription Kit (Takara, Osaka, Japan). The following PCR process was performed on a Real-Time System (ABI 7500, Applied Biosystems, Carlsbad, California, USA) using a SYBR Green kit (Roche, Basel, Switzerland). Primer sequences are listed below.

- GAPDH: (F: 5’-GAG TCC TTC CAC GAT ACC AA-3’ and R: 5’-ACG TCG CAC TTC ATG ATC GAG-3’);
- circSNX27: (F: 5’-AAA AGC AAG CAG TGC CCA TA-3’ and R: 5’-GCT CGA ATC AGG TCC ACC A-3’);
- MiR-638: (F: 5’-GAGGCACATAACCTAGATCCCAG-3’ and R: 5’-TCCGGCGGTGGGCGGGCGCTAGGGA-3’);
- MiR-581: (F: 5’-TGTTGAGGGGGCGACACACAAGC-3’ and
- R: 5’-TGACTAGATCTCTTGTGTTCT-3’);
- MiR-769-3p: (F: 5’-GAGGCAGATAACCTAGATCCCAG-3’ and
- R: 5’-TTGGTTCTGGGGCCTCTAGGGTC-3’).

### Ribonucleic Acid Stability Assay

The stabilization of circSNX27 was explored by actinomycin D. circSNX27 and SNX27 mRNA in HGC27 cells were tested by qRT-PCR after treatment with actinomycin D (5 µg/mL) for 24 hours.

### Plasmid Construction and Transfection

CircSNX27-specific short hairpin RNA (shRNAs) (shcircSXN27-1, shcircSXN27-2, and shcircSXN27-3) were established by placing circSNX27 sequence into a lentivirus vector (pGLVH1, GenePharm, China). Transfected into HEK293T cells, and stably expressed cells were screened by puromycin (2 μg/mL). circSNX27 overexpression plasmid and miR-638 mimics, as well as their negative controls, were supplied by Hanheng Biotech (Shanghai, China). 

### Cell Viability, Proliferation, and Apoptosis Assessment

For the CCK-8 assay, GC cells (1 × 10^5^ cells) were gathered after the indicated treatment and plated into 96-well plates with 150 µL culture medium. Cells were cultured overnight at 37°C, and then 10 µL of CCK-8 solution was added (Dojindo, Kumamoto, Japan) into each well. After another 2 hours of incubation, a microplate reader (BioRad, Hercules, California, USA) was used to measure the absorbance at 450 nm. For colony formation assay, GC cells (1 × 10^4^ cells) were gathered after indicated treatment and plated into 6-well plates. After 2 weeks of culture, the cell colonies were fixed by methanol and visualized by 0.5% crystal violet. Colonies were manually counted under a microsope (Olympus, Tokyo, Japan). For cell apoptosis assessment, treated GC cells were collected and fixed with 70% pre-cold ethanol. Cells were then incubated with propidium/annexin V-FITC (Sigma-Aldrich, St. Louis, Missouri, USA) for 20 minutes after washing with PBS for 3 times. Cell apoptosis rate was next assessed through a flow cytometer (Gallios, Brea, California, USA).

### Cell Migration and Invasion Assessment

Wound-healing assay was adopted to assess the migration of treated HGC27 cells. In brief, treated HGC27 cells were gathered and seeded in 6-well plates with a density of 5 × 10^5^ cells per well. A wound was then made by a sterile pipette tip on the cell monolayer after it reached 100% confluence. Photographs were taken after 0 and 24 hours of the scratching. Transwell chambers with Matrigel matrix (Corning, USA) were employed to test the invasion ability of HGC27 cells. Treated HGC27 cells (5 × 10^4^) in culture medium with no FBS were placed into the upper chamber, and the lower chamber was filled by culture medium containing 10% FBS. After 24 hours of incubation, fixed the cells on the backside of polycarbonate film with 4% methanol, followed by staining with 0.1 crystal violet. Cells in 5 randomly selected view fields were counted manually.

### Luciferase Reporter Assay

The wild-type or mutant circSNX27 sequence containing miR-638 binding site was inserted into a luciferase vector, pGL3 (Genechem, China) to establish luciferase reporter plasmid and named as circSNX27-WT or circSNX27-MUT. circSNX27-WT or circSNX27-MUT were co-transfected into HGC27 cells with miR-638 mimics or miR-NC using lipofectamine 3000 reagent (Invitrogen). Next, the luciferase activity was detected by a luciferase assay kit (Promega, Madison, Wisconsin, USA) after 48 hours of co-transfection.

### Statistical Analysis

Graphpad Prism (Version 9.0, USA) was adopted for data statistical analyses. Data were organized as mean ± SD, and all experiments were performed in triplicate. The Student’s *t*-test was used to compare differences between sh-NC group and shcircSNX27 group. The paired Wilcoxon test was used to compare the expression level between gastric adenocarcinoma and corresponding normal tissues. For all analyses, *P*-values < .05 from 2-tailed tests were considered statistically significant.

## Results

### circSNX27 Expression is Elevated in Gastric Cancer

**Figure 1.**
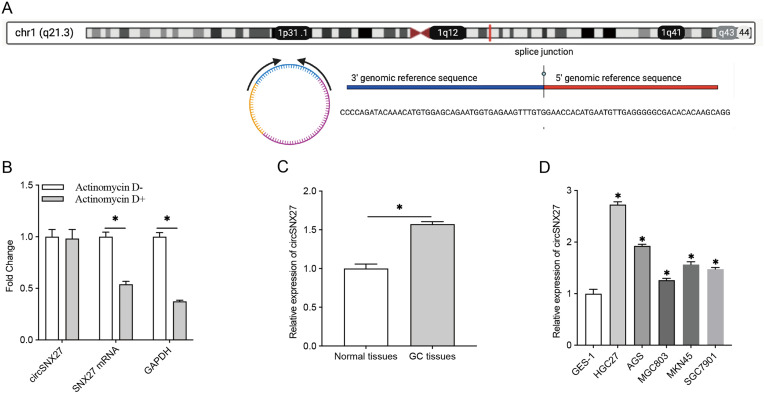
circSNX27 expression is elevated in GC. (A) circSNX27 arises from SNX27 gene, which is located at chromosome 1 (chr1:151611363-151611595). (B) Expression levels of circSNX27 and SNX27 mRNA in HGC27 cells were tested by qRT-PCR after treatment with actinomycin D (5 µg/mL) for 24 hours. (C) qRT-PCR analysis of circSNX27 expression level in 26 pairs of normal and GC tissues. (D) qRT-PCR analysis of circSNX27 expression level in 5 GC cell lines, HGC27, AGS, MGC803, MKN45, and SGC7901. GES-1 was used as control. GC, gastric cancer; mRNA, messenger RNA; qRT-PCR, quantitative reverse transcription polymerase chain reaction.

As shown in Figure 1A, circSNX27 arises from the SNX27 gene, which is located at chromosome 1 (chr1:151611363-151611595). To confirm the circular structure of circSNX27, the stabilizing of circSNX27 was explored by actinomycin D treatment. Then, qRT-PCR method was performed to detect the expression of SNX27 mRNA and circSNX27 after 24 hours of treatment with actinomycin D (5 µg/mL). We found that circSNX27 was more stable than linear SNX27 mRNA in GC cells under actinomycin D treatment (Figure 1B). To determine whether circSNX27 promotes GC tumorigenesis, we examined the expression level of circSNX27 in GC tissues and corresponding normal tissues. It was found that circSNX27 was upregulated in the cancer group (Figure 1C). We also detected circSNX27 expression in 5 GC cell lines: HGC27, AGS, MGC803, MKN45, and SGC7901. Compared to GES-1, circSNX27 was dramatically increased in the 5 GC cell lines, and HGC-27 showed the highest circSNX27 expression (Figure 1D). These results suggested that circSNX27 might play a role in GC tumorigenesis.

### Silencing circsnx27 Represses Gastric Cancer Cell Proliferation Viability and Promoted Gastric Cancer Cell Apoptosis

**Figure 2.**
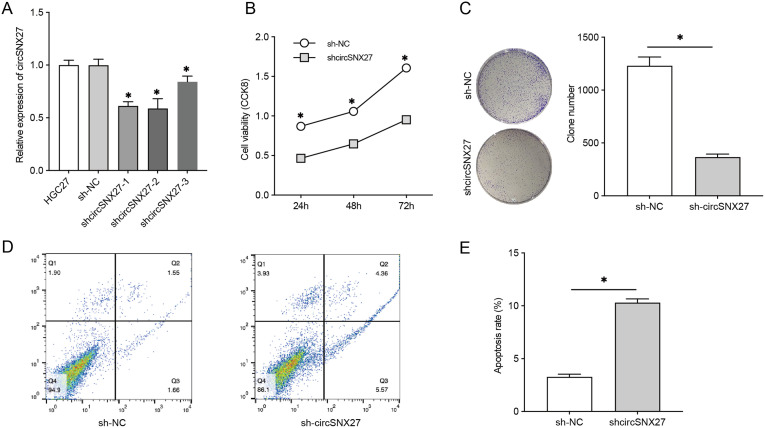
Silencing of circSNX27 represses GC cell proliferation viability and promoted GC cell apoptosis. (A) Three shRNAs were designed to silence the expression of circSNX27, and their knockdown efficiency was tested in HGC27 cells. (B) Viability of HGC27 cells was examined by CCK-8 at 24 hours, 48 hours, and 72 hours after treatment with sh-NC and sh-SNX27. (C) Proliferation activity of HGC27 cells was tested by clone formation assay after 48 hours of treatment with sh-NC or sh-SNX27. (D) Representative images of flow cytometry analysis of apoptosis rats of sh-NC and sh-circSNX27-treated HGC27 cells. (E) Apoptosis of HGC27 cells was tested by flow cytometry analysis after 24 hours of treatment with shSNX27. GC, gastric cancer; shRNA, short hairpin RNA.

To determine the functional role of circSNX27 in GC, 3 specific circSNX27 shRNAs (shcircSNX27-1, shcircSNX27-2, and shcircSNX27-3) were designed to silence circSNX27 expression, and the impacts of circSNX27 knockdown on cell viability, proliferation, and apoptosis were estimated. Compared to the sh-NC group, circSNX27 expression was sharply decreased in shcircSNX27-1, shcircSNX27-2, and shcircSNX27-3 groups (Figure 2A). Thus, we used a mixture of the 3 shRNAs in the following functional assays and named as shcircSNX27. CCK-8 analysis showed that shcircSNX27 transfection repressed HGC27 cell viability compared with shNC-transfected cells (Figure 2B). In colony formation assay, we found a remarkable reduction of colony number in shcircSNX27-transfected HGC27 cells (Figure 2C), suggesting that circSNX27 silencing repressed HGC27 cell proliferation. Moreover, in the flow cytometry apoptosis analysis, an elevation of cell apoptosis was observed after transfection with circSNX27 (Figure 2D and 2E). These findings indicated that circSNX27 silencing reduced cell viability, suppressed cell proliferation, and facilitated cell apoptosis in GC cells.

### Silencing circsnx27 Represses Gastric Cancer Cell Migration and Invasion

**Figure 3.**
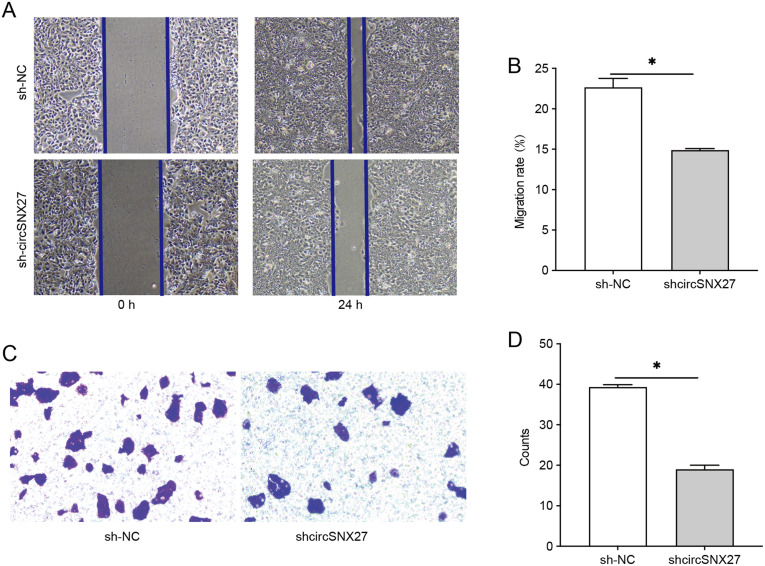
Silencing of circSNX27 represses GC cell migration and invasion. (A) Representative images of wound-healing assay. (B) Statistical analysis of migration rate of HGC cells treated with sh-NC and sh-circSNX27. (C) Representative images of invasive HGC27 cells from sh-NC and sh-circSNX27 group. (D) Statistical analysis of the invasive ability of HGC27 cells after treatment with sh-NC and sh-circSNX27. GC, gastric cancer.

We next tested the influence of circSNX27 silencing on HGC27 cells migration and invasion in vitro. The results indicated that the wound-healing speed was significantly slower in shcircSNX27 group compared to shNC group, suggesting that circSNX27 silencing repressed HGC27 cell migration (Figure 3A and 3B). Also, results of transwell assays suggested that shcircSNX27 reduced the invasion of HGC27 cells (Figure 3C and 3D). These results indicated that circSNX27 silencing inhibited HGC27 cell migration and invasion.

### circSNX27 Binds to and Negatively Regulates miR-638

**Figure 4.**
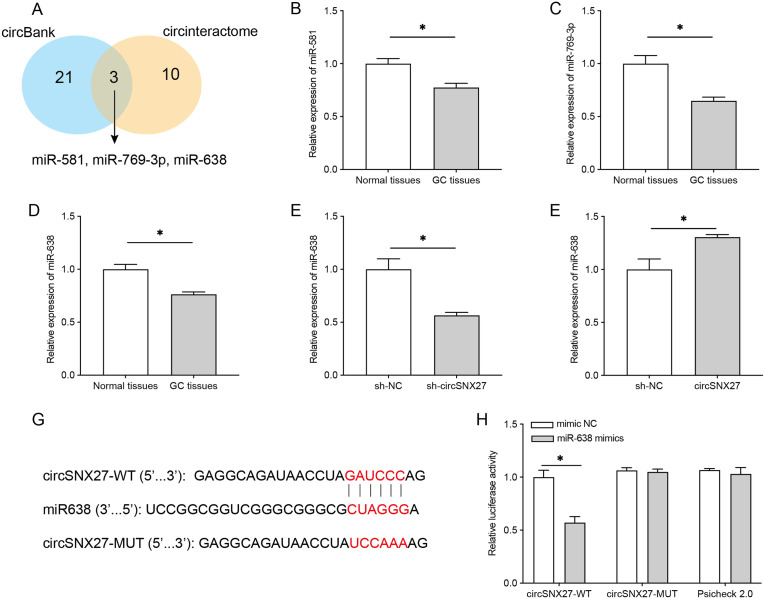
circSNX27 binds to and negatively regulates miR-638. (A) The 3 target miRNAs of circSNX27 (miR-581, miR-769-3p, and miR-638) were identified based on the overlap of prediction results from circBank and circinteractome. (B-D) Relative expression levels of miR-581, miR-769-3p, and miR-638 in normal and GC tissues were tested by qRT-PCR. (E and F) Relative expression level of miR-638 in HCG27 cells treated with sh-circSNX27 or circSNX27. (G) The putative sequences with wild-type or mutant miR-638 binding sites. (H) Interaction between circSNX27 and miR-638 was tested by dual-luciferase reporter assay. GC, gastric cancer; qRT-PCR, quantitative reverse transcription polymerase chain reaction.

The above results suggest that circSNX27 acts as an oncogene of GC in vitro; however, the underlying mechanisms remain unclear. To answer this question, we screened the target miRNAs of circSNX27 based on the prediction results of circBank and Circinteractome bioinformatics databases. Three miRNAs (miR-581, miR-769-3p, and miR-638) were identified in both databases (Figure 4A). To determine whether these miRNAs play a role in GC tumorigenesis, we tested their expression in GC tissues. Compared to normal controls, relative expression of miR-581, miR-769-3p, and miR-638 were dramatically decreased in GC tissues (Figure 4B-4D). Herein, miR-638 was selected for further study. Relative expression of miR-638 was detected in circSNX27-overexpressed and silenced HGC27 cells. Overexpression of circSNX27 caused a remarkable reduction of miR-638 (Figure 4E), while silencing of circSNX27 significantly increased the expression of miR-638 (Figure 4F). To verify the interplay between circSNX27 and miR-638, dual-luciferase reporter assay was performed. Transfection of miR-638 mimics could reduce the luciferase activity of HGC27 cells driven by circSNX27-WT but not circSNX27-MUT (Figure 4G and 4H). These results indicated that circSNX27 negatively regulates the expression of miR-638 in GC by directly binding to it.

## Discussion

Numerous studies have proved that circRNA expression profile is changed in GC;^12^ however, the exact role of each circRNA in GC tumorigenesis has not been well investigated due to the large number of circRNAs. Some of the circRNAs act as oncogenes for GC,^13,14^ while others act as repressors for GC.^15,16^ Thus, the “net effects” of circRNAs on GC cannot be explained without understanding the role of each related circRNAs. For this study, we studied the role of circSNX27 in GC tumorigenesis.

circSNX27 was previously demonstrated to be elevated in HCC by several studies, and multiple molecular mechanisms were found. For instance, circSNX27 was found to promote HCC progression as a ceRNA factor.^11^ Another molecular mechanism was discovered by Li et al^17^; their findings suggested that circSNX27 contribute to the development of HCC through controlling miR-637/FGFR1 axis. These findings suggested that circSNX27 could regulate the progression of HCC through multiple different mechanisms. However, the role of circSNX27 in GC remains undetermined. Herein, for the first time, we reported that circSNX27 was dramatically elevated in GC patients’ tissues and cell lines. Moreover, circSNX27 was stable in GC and resistant to the treatment of actinomycin D, indicating that it could be used as a promising biomarker of GC diagnosis. In addition, the circSNX27 knockdown experiments suggested that circSNX27 silencing could inhibit GC cell proliferation, invasion, migration, and increase the apoptosis of GC cells. Although the results need to be confirmed by further *in vivo* evidence, it still suggests that circSNX27 repression may function in the treatment of GC patients.

circRNAs are well known to affect the expression and functions of target genes by acting as a sponge of miRNAs^18^; thus, 2 databases (circBank and Circinteractome) were used to screen the target miRNAs of circSNX27. A total of 3 miRNAs (miR-581, miR-769-3p, and miR-638) were identified in both databases, and they were all found to be decreased in GC tissues, suggesting they may work in GC development. The expression of miR-769-3p was found to be decreased before by Dai et al^19^; they found that miR-769-3p overexpression could repress GC malignant biological behaviors through the STAT3–IGF1R–HDAC3 complex. For miR-581, it was found to promote the progression of colorectal cancer, liver cancer, and prostate cancer.^20-22^ However, its role in GC has not been investigated yet. For miR-638, it has been well documented to be a suppressor of GC.^23-25^ Due to limited funds, we only tested the interaction between circSNX27 and miR-638, and we found that circSNX27 negatively regulates the expression of miR-638 by directly binding to it.

In summary, these findings suggested that circSNX27 involves in the GC progression regulation through acting as a sponge of miR-638. However, circSNX27 may also be involved in the regulation of GC through the other 2 miRNAs and their target genes.

## Data Availability

Data will be made available on request.
